# Nasal hemophilic pseudotumor in a 2-year-old with recurrent epistaxis: a case report and review of the literature

**DOI:** 10.1093/jscr/rjad586

**Published:** 2023-11-03

**Authors:** Nouran Farag, Bashair Alwasiyah, Osama Marglani

**Affiliations:** Department of Otolaryngology—Head & Neck Surgery, Dr. Soliman Fakeeh Hospital, Jeddah, Saudi Arabia; Department of Otolaryngology—Head & Neck Surgery, King Fahad General Hospital, Jeddah, Saudi Arabia; Department of Otolaryngology—Head & Neck Surgery, King Faisal Specialist Hospital and Research Center, Jeddah, Saudi Arabia; Department of Otolaryngology—Head & Neck Surgery, Umm Al-Qura University, Makkah, Saudi Arabia

**Keywords:** epistaxis, hemophilia, nasal, pseudotumor, endoscope

## Abstract

Hemophilic pseudotumor is a rare, yet dangerous complication of hemophilia. It has been reported previously at sites prone to recurrent trauma like long bones and pelvis. However, in the field of otorhinolaryngology, few cases are reported and therefore there is no established protocol for management. We hereby report a case of a 2-year-old boy, a known case of hemophilia A (factor VIII deficiency), who presented with recurrent epistaxis not responding to medical management. Imaging was done and revealed a heterogenous nasal mass compressing the left orbital wall, extending to the sphenoid sinus, and causing skull base erosion. The patient was successfully managed by evacuation and drainage of the pseudotumor via endoscopic endonasal approach and replacement of factor VII pre-and post-operatively. To our knowledge, this is the first case of nasal hemophilic pseudotumor managed by evacuation and drainage through an endoscopic endonasal approach, which was deemed successful.

## Introduction

A pseudotumor is a recurrent chronic muscle hematoma. In patients with hemophilia, it can become a hemophilic pseudotumor, which is a rare, unusual, but serious complication of hemophilia [1]. It is a slowly progressive encapsulated cystic mass that occurs at sites of recurrent hemorrhage, usually in soft tissue, and less commonly in bone or a subperiosteal location [2].

## Case report

This patient is a 2-year-old boy, a known case of Hemophilia A (factor VIII deficiency) diagnosed when he was 1 month old. He had recurrent spontaneous epistaxis for 6 months, not precipitated by trauma; and presented to the emergency department complaining of bleeding from the left nostril that did not stop even after tranexamic acid administration. There was no history of bleeding from other sites. On examination, the boy was active and hemodynamically stable. There was blood oozing from the nose and a noticeable left eye deviation with mild proptosis.

**Figure 1:**
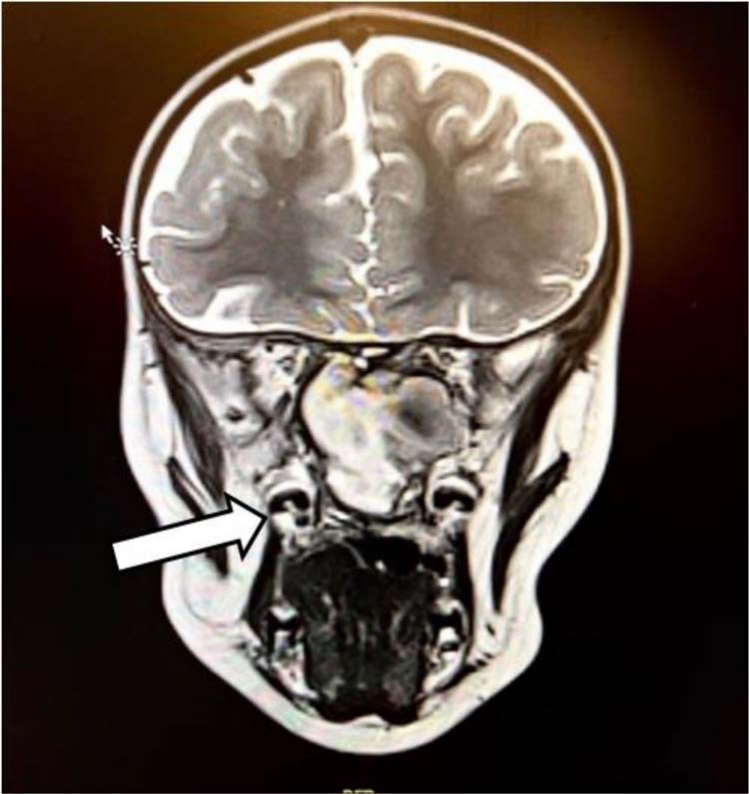
The coronal section of the MRI.

**Figure 2:**
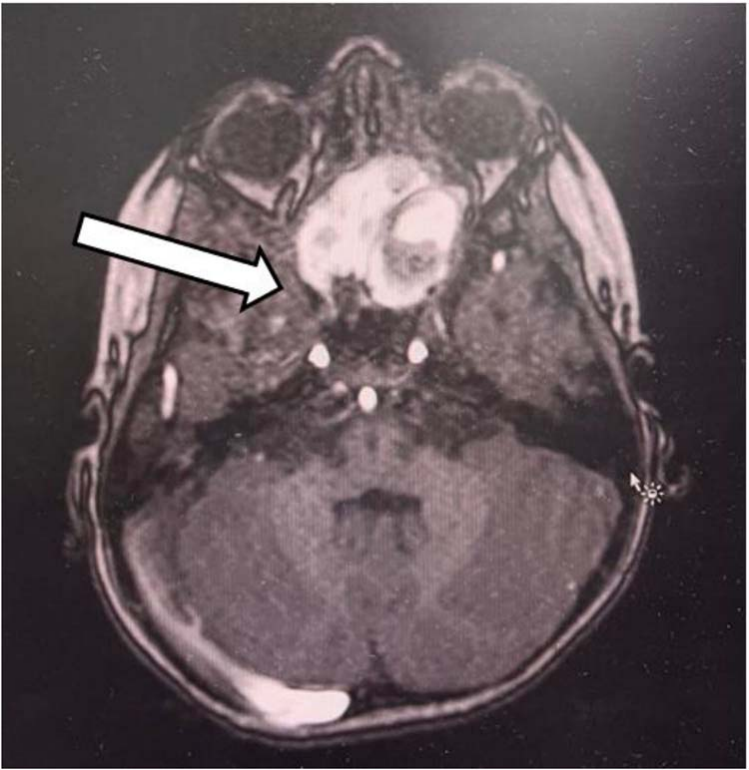
The axial section of the MRI.

The patient had undergone computed tomography of the paranasal sinuses (CT PNS) and magnetic resonance imaging (MRI) brain with contrast. The MRI showed a heterogeneous mass predominantly occupying the left nasal cavity, left maxillary, and sphenoid sinus, destructing the osseous structure, as well as extending to the medial aspect of the left orbital wall causing minimal proptosis (Figs 1 and 2). Magnetic resonance angiography showed no prominent feeding vessel of the mass. CT PNS demonstrated a heterogenous soft tissue nasal mass extending into the sphenoid wing through the sphenoid sinus, causing skull base erosion but no intracranial invasion.

The decision to intervene via endoscopic endonasal approach (EEA) for evacuation and drainage of the pseudotumor was taken by otorhinolaryngologists. The patient was admitted for elective surgery, and pre-operative optimization by replacement of factor VII (NovoSeven) was done because he had inhibitors to factor VIII, which he was previously on.

**Figure 3:**
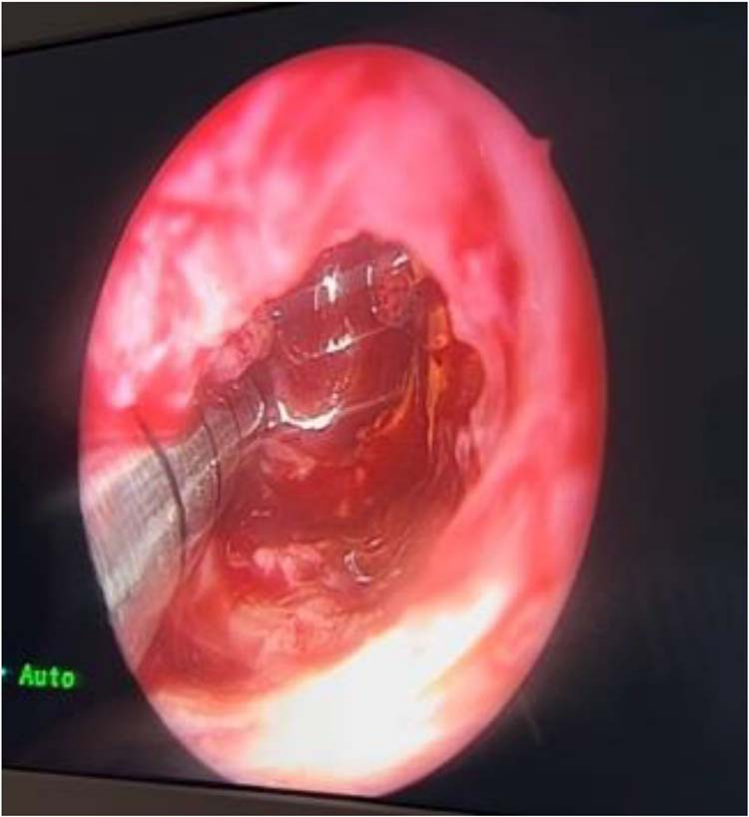
Evacuation of the mass.

**Figure 4:**
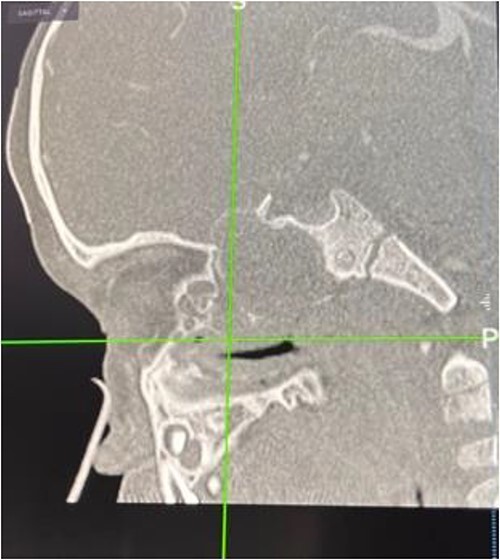
Skull base erosion on CT navigation.

**Figure 5:**
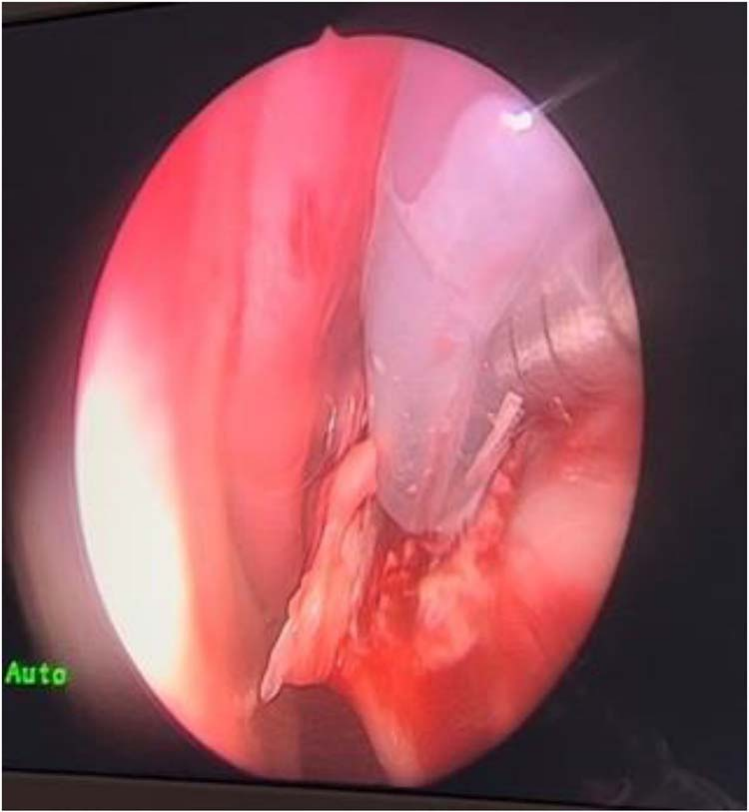
Doyle splint inserted.

In the operating room (OR), examination under general anesthesia (GA) using a rigid nasal endoscope showed a heterogenous mass in the posterior nasal septum destructing the vomer, rostrum, and sphenoid wing. The mass was evacuated by the EEA while using the navigation system. The evacuation extended posteriorly to the sphenoid sinus, laterally to the sphenoid wing, and superiorly to the skull base (Figs 3 and 4). Lastly, Doyle nasal splints were inserted bilaterally to prevent re-accumulation of a hematoma (Fig. 5). Post-operatively, NovoSeven was administered for optimization. The patient was discharged in stable condition, and 10 days later underwent removal of the splints in the OR under GA. He followed up post-operatively at the otolaryngology clinic and was doing well, with no further complaints of epistaxis.

## Discussion

Even though it is a rare entity, pseudotumor is a dangerous complication of hemophilia. The etiology is believed to be related to sustaining repeated trauma [3]. According to Kwon *et al.*, only 37.5% of patients with hemophilic pseudotumor had a history of trauma. Other etiologies included mumps, incisional biopsies, and extractions [4]. Ogata *et al.* reported a case of a boy diagnosed with nasal hemophilic pseudotumor, who frequently compressed his nose using his hand due to allergic rhinitis [2]. This suggests that even indirect trauma can predispose to recurrent bleeding and lead to the formation of nasal soft tissue mass.

Hemophilic pseudotumor has been shown to occur at different sites, most commonly in the long bones of the lower extremities and pelvis [1]. This can be attributed to accidental falls while walking. Other sites that are less commonly involved include the cranium [5], jaw [2], orbit [6], tibia [7], and small bones of hands [8].

The diagnosis of hemophilic pseudotumor can be challenging. Diagnostic modalities like percutaneous aspiration and needle biopsies can determine the histopathological nature of the lesion but, according to Purkait *et al.*, are discouraged due to the high risk of complications like infection, hemorrhage, and fistulization [3]. On the contrary, Rodriguez-Merchan *et al.* highlighted the importance of biopsies to confirm the diagnosis and rule out true tumors like liposarcoma, chondrosarcoma, and synovial sarcoma [9]. In cases where hemophilia is established, high-quality CT scans and MRI are believed to be the best diagnostic modalities [3, 4, 10].

There is no standard protocol for managing cases of hemophilic pseudotumor. Different methods, like radiotherapy, surgical intervention, and factor VIII replacement have been reported in the literature. Each method has its advantages and limitations. The first case of nasal hemophilic pseudotumor in 1997, at Queen Elizabeth Hospital, Birmingham, was successfully managed by debulking of the prominent lesion on the nasal dorsum [1]. According to Purkait *et al.*, a 3-year-old boy with nasal hemophilic pseudotumor improved dramatically on radiotherapy and the size of the mass decreased gradually. However, there is no standard radiation dose or fractionalization schedule to be implemented [3]. Ogata *et al*. reported a case of nasal hemophilic pseudotumor that was successfully managed conservatively only by factor VIII concentrates and repeat CT for two consecutive years showed complete recovery of the surrounding bone of the nose [2]. Even though factor VIII can be effective, it poses a challenge in patients with inhibitors to it, which was the case for our patient. Similarly, Mandal *et al*. reported a case of nasal hemophilic pseudotumor in an infant with severe hemophilia A and high titer inhibitors to factor VIII; therefore, the patient underwent surgical excision [11]. Another case of nasal hemophilic pseudotumor was reported by Sulochana *et al*. in which the patient had recurrent unprovoked epistaxis and benefited from both surgery and factor VIII replacement [12]. Surgical intervention is considered a reasonable option yet poses a challenge for surgeons when the site is relatively inaccessible, like the case reported by Gupta *et al*. for a hemophilic pseudotumor in the ethmoid and sphenoid paranasal sinuses which was rather managed by radiotherapy and factor VIII concentrates [13] Another possible option, according to Rodriguez Merchan *et al*., is preoperative arterial embolization which may be helpful in cases of large pelvic pseudotumors [9].

## Conclusion

Hemophilic pseudotumor is a dangerous complication of hemophilia, yet rarely reported in the field of otolaryngology. We reported this case of a 2-year-old boy with nasal hemophilic pseudotumor to increase the awareness of the otolaryngologists’ community regarding these rare entities, which we found to be successfully managed by evacuation and drainage through EEA.

## Data Availability

We hereby confirm that all presented data that requires identification was correctly and appropriately cited in the references section.
